# Peptide toxins that target vertebrate voltage-gated sodium channels underly the painful stings of harvester ants

**DOI:** 10.1016/j.jbc.2023.105577

**Published:** 2023-12-16

**Authors:** Samuel D. Robinson, Jennifer R. Deuis, Pancong Niu, Axel Touchard, Alexander Mueller, Vanessa Schendel, Nina Brinkwirth, Glenn F. King, Irina Vetter, Justin O. Schmidt

**Affiliations:** 1 Institute for Molecular Bioscience, The University of Queensland, Queensland, Australia; 2 CNRS, UMR Ecologie des forêts de Guyane – EcoFoG (AgroParisTech, CIRAD, INRAE, Université de Guyane, Université des Antilles), Kourou, France; 3 Centro de Investigación Biomédica CENBIO, Universidad UTE, Quito, Ecuador; 4 Nanion Technologies, Munich, Germany; 5 Australian Research Council Centre of Excellence for Innovations in Peptide and Protein Science, The University of Queensland, Brisbane, Queensland, Australia; 6 School of Pharmacy, The University of Queensland, Brisbane, Queensland, Australia; 7 Southwestern Biological Institute, Tucson, Arizona, USA

**Keywords:** venom, *Pogonomyrmex*, ion channel, pain, neuron

## Abstract

Harvester ants (genus *Pogonomyrmex*) are renowned for their stings which cause intense, long-lasting pain, and other neurotoxic symptoms in vertebrates. Here, we show that harvester ant venoms are relatively simple and composed largely of peptide toxins. One class of peptides is primarily responsible for the long-lasting local pain of envenomation *via* activation of peripheral sensory neurons. These hydrophobic, cysteine-free peptides potently modulate mammalian voltage-gated sodium (Na_V_) channels, reducing the voltage threshold for activation and inhibiting channel inactivation. These toxins appear to have evolved specifically to deter vertebrates.

One defining feature of the aculeate Hymenoptera is the capacity of many species to deliver a painful defensive sting. The most familiar stings, such as those of the eusocial bees of the genus *Apis* or eusocial wasps of the family Vespidae, typically cause intense but short-lasting pain (<10 min), and subsequent redness, swelling, and itch ((1) and author observations S. D. R and J. O. S). In the case of *Apis* envenomation, the “short, sharp” pain is thought to be primarily mediated by melittin, a membrane-active amphipathic venom peptide (2, 3). This venom mode-of-action is thought to be shared by other hymenopterans that cause similar envenomation symptoms in humans (3, 4, 5, 6). However, envenomation by certain lineages of hymenopterans can produce symptoms in humans that deviate from this “norm” (1) and is suggestive of the evolution of distinct defensive venom chemistry. An extreme example of this can be observed in certain ants, particularly the harvester ants of the genus *Pogonomyrmex*.

*Pogonomyrmex* are distributed across parts of North and South America as well as the Caribbean island of Hispaniola. They are thought to use their sting solely for defense. Indeed, species of the *californicus* group of North American *Pogonomyrmex* (*e.g. Pogonomyrmex maricopa*; Fig. 1*A*), can autotomize their stings (Fig. 1*B*) (7), an adaptation strictly associated with defense. In humans, *Pogonomyrmex* stings can cause intense local pain that, while slowly developing (typically 1–2 min), can last for several hours and is accompanied by localized piloerection, swelling, axon reflex flare, and extreme hyperhidrosis ((1, 8, 9) and author observations S. D. R and J. O. S; Fig. 1*C*).

**Figure 1:**
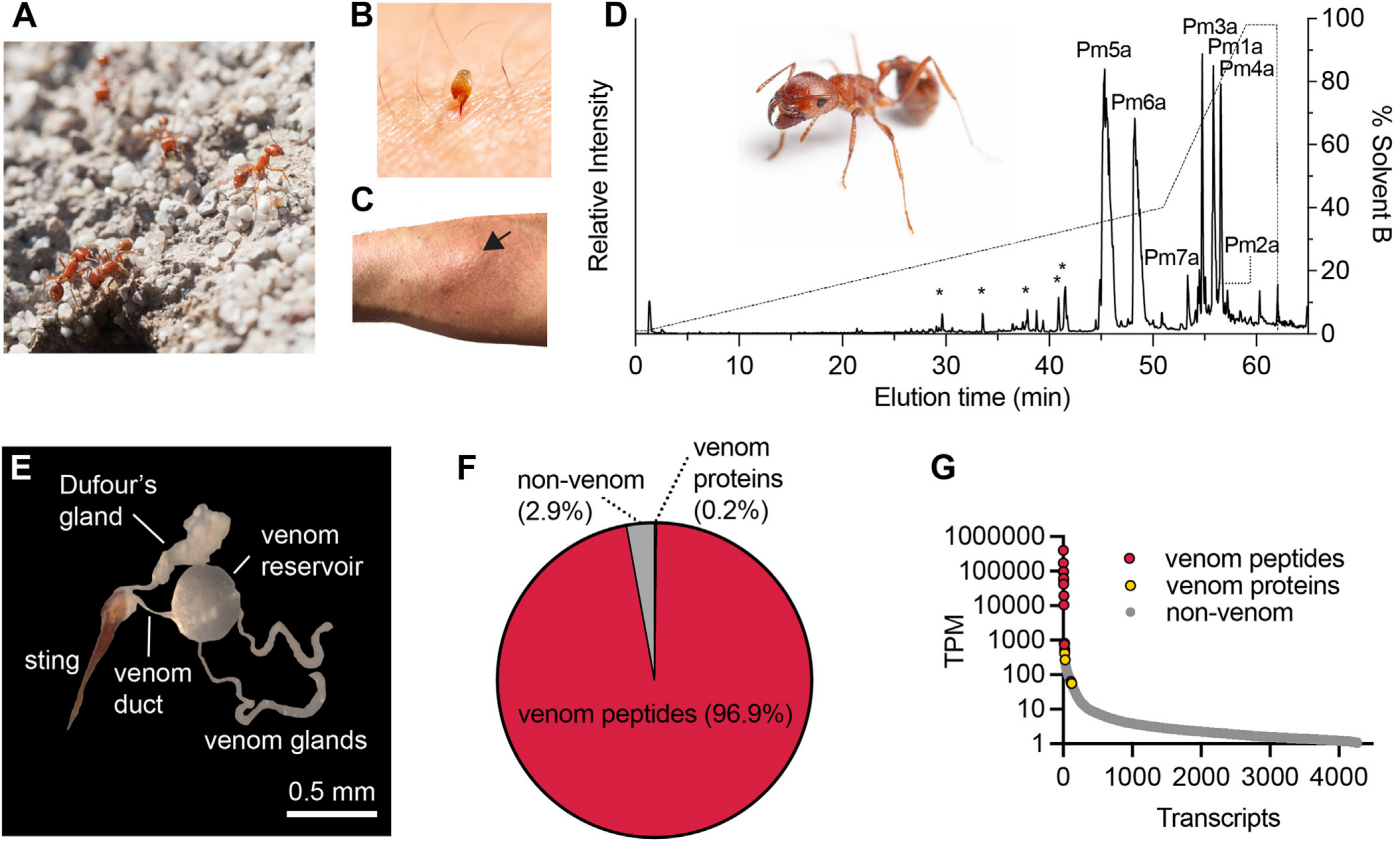
**The venom of *Pogonomyrmex maricopa*.***A*, *P. maricopa* adult female worker caste (∼8 mm in length) at nest entrance. *B*, sting autotomy occurs in several *Pogonomyrmex* species including *P. maricopa*. Shown here is an autotomized sting (∼2 mm) of *P. maricopa*, embedded in a human forearm (S. D. R) *C*, human forearm (S. D. R). ∼30 min after being stung, illustrating local piloerection, swelling, hyperhidrosis, and axon reflex flare. The site of envenomation is indicated by the arrow. *D*, total ion chromatogram from LC-MS/MS analysis of *P. maricopa* venom with labeled peaks corresponding to identified venom peptides (venom peptide sequences are shown in Table 1). Additional proteins were detected in the reduced, alkylated, and trypsin-digested venom sample. *Asterisks* mark derivatives of identified venom peptides. *Inset*: *P. maricopa* adult female worker (∼7 mm in length). *E*, venom apparatus of *P. maricopa*. *F*, venom component-encoding transcripts (*i.e.*, those encoding peptides and proteins detected in the venom itself) comprised 97.1% of total venom gland transcriptome expression. Of these, transcripts encoding venom peptides and venom proteins constituted 96.9% and 0.2%, respectively. *G*, venom component-encoding transcripts (highlighted in *red*) are found exclusively in the highly expressed portion of the venom apparatus transcriptome. LC-MS/MS, liquid chromatography-tandem MS; TPM, transcripts per million.

*Pogonomyrmex* venoms have the greatest lethality to mammals of any insect venom tested to-date (9, 10). *Pogonomyrmex barbatus* venom administered intraperitoneally in mice causes death associated with clonic convulsions with an LD_50_ of 1.9 mg/kg (8). The venoms of several other *Pogonomyrmex* species are more potent, *e.g.* that of *P. maricopa* is approximately 10-fold more potent than that of *P. barbatus* in the same assay, with an LD_50_ of 0.12 mg/kg (7, 11).

Together, these observations have led some to hypothesize the existence, in *Pogonomyrmex* venoms, of one or more potent neurotoxins (7, 9, 10, 12). In this study, we uncover the chemistry and pharmacology underpinning the characteristic long-lasting local pain of *Pogonomyrmex* envenomation.

## Results

We used a combined transcriptomic and mass spectrometry (MS)-based strategy to generate a full profile of the venom composition of *P. maricopa*. RNA extracted from the venom-apparatus (venom gland filaments, venom reservoir, and venom duct; Fig. 1*E*) dissected and pooled from ∼20 workers, was used to generate a venom apparatus transcriptome. We obtained 15,028,344 demultiplexed paired-end reads from Illumina NextSeq RNA sequencing, which, following adaptor trimming, quality trimming, and filtering and error correction, were assembled *de novo* using Trinity to yield a total of 4624 contigs. We collected *P. maricopa* venom by dissecting out venom reservoirs from ∼20 workers, squeezing the contents into water and pooling. Liquid chromatography-tandem MS data from three venom samples (native; reduced and alkylated; reduced, alkylated, and trypsin-digested) were searched against a database comprising the translated venom-apparatus transcriptome.

Analysis of the venom of *P. maricopa* (native or reduced and alkylated) by LC-MS indicated that it was relatively simple (Fig. 1*D*). The major peaks in the total ion chromatogram of the native venom corresponded to seven peptides which we named Myrmicitoxin_1_-(MYRTX_1_)-Pm1a, MYRTX_1_-Pm2a, MYRTX_1_-Pm3a/b, MYRTX_1_-Pm4a/b, MYRTX_1_-Pm5a, MYRTX_1_-Pm6a, and MYRTX_1_-Pm7a/b (Table 1). Pm3a/b, Pm4a/b and Pm7a/b were so named because for each, two paralogous transcripts encoded the same mature peptide. One additional sequence, MYRTX_1_-Pm8a, was not detected in our LC-MS data but had high-sequence similarity and a similarly high-expression level estimate to the other identified venom peptides. Transcripts encoding these peptides accounted for 96.9% of the venom apparatus transcriptome reads (Fig. 1, *F* and *G*). Of these, the transcript encoding Pm1a was the most highly expressed, accounting for almost half of the total venom-apparatus transcriptome (401,155 transcripts per million).

**Table 1:** Venom components of *Pogonomyrmex maricopa*

Venom components	TPM	Primary structure
Venom peptides		
Pm1a	401155	GLPLLALLFSLPVLQHWIEKNWIN^a^
Pm2a	10454	ALPALPLLMFLFTLPALQHWIEKNWIN^a^
Pm3a/b	191152^b^	GLPILALFVTIPFIHHYLMEKL^a^
Pm4a/b	104455^b^	LLPLIPILASLIAAIKS^a^
Pm5a	170748	IDLKQIMEKVKPDLLKMLDDIKAKIQQ^a^
Pm6a	53531	GIFSALKTLGKILLPVILPTVAEKIKEKV^a^
Pm7a/b	1616^b^	ADKPGQAKEIGIFDRITELINWLVNH
Pm8a^c^	19596	
Enzymes		
Acid phosphatase^d^	1150	
PLA_1_^d^	723	
DPP-IV^d^	57	
Endochitinase^d^	44	
PHGPX^d^	22	
PPI^d^	19	
Esterase^d^	5	

TPM, transcripts per million

a *C*-terminal amidation

b The TPM value is the sum of two paralogous transcripts encoding the same mature peptide.

c No mature peptide detected in the venom

d Detected in reduced, alkylated, trypsin-digested venom sample (>60% coverage), sequences not shown; PLA_1_, phospholipase-A_1_; DPP-IV, dipeptidyl peptidase IV; PHGPX, Phospholipid-hydroperoxide glutathione peroxidase; PPI, Peptidyl-prolyl *cis-trans* isomerase.

Several enzymes were detected by bottom-up sequencing of the reduced, alkylated, and trypsin-digested venom sample. These included acid phosphatase, phospholipase A_1_ (PLA_1_), dipeptidyl peptidase IV (DPP-IV), phospholipid-hydroperoxide glutathione peroxidase (PHGPX), and a peptidyl-prolyl *cis-trans* isomerase. These accounted for 0.2% of the venom apparatus transcriptome reads (Fig. 1, *F* and *G*). All of these enzymes, with the exception of PHGPX, have previously been reported in hymenopteran venoms. In *P. maricopa* venom, PLA_1_ presumably catabolizes phospholipids in cell membranes, possibly enhancing the efficacy or spread of other venom components (3, 13), DPP-IV likely plays a role in venom peptide maturation, while the function of the other enzymes in the venom remains unclear.

Previous studies have suggested that the venom composition of different *Pogonomyrmex* species are likely to be similar (7, 14, 15). To test this hypothesis, we investigated the venom composition of *Pogonomyrmex rugosus* using the same strategy as for *P. maricopa*. For *P. rugosus*, we identified a total of 13 venom peptides, which together accounted for 88.0% of the *P. rugosus* venom-apparatus transcriptome reads (Fig. S1; Table S1). Twelve of the thirteen peptides had homologues in *P. maricopa* venom. Several proteins, which accounted for 4.9% of venom apparatus transcriptome reads, were detected by bottom-up sequencing of the reduced, alkylated and trypsin-digested venom sample (Fig. S1; Table S1). These included four of the six enzymes detected in *P. maricopa* venom: acid phosphatase, PLA_1_, DPP-IV, and PHGPX. In addition, we detected transcripts encoding CAPs (cysteine-rich secretory protein, insect venom allergen antigen 5, and pathogenesis-related 1 proteins) and hyaluronidase. These data indicated that the venoms of *P. rugosus* and *P. maricopa* share a high degree of similarity.

**Figure 2:**
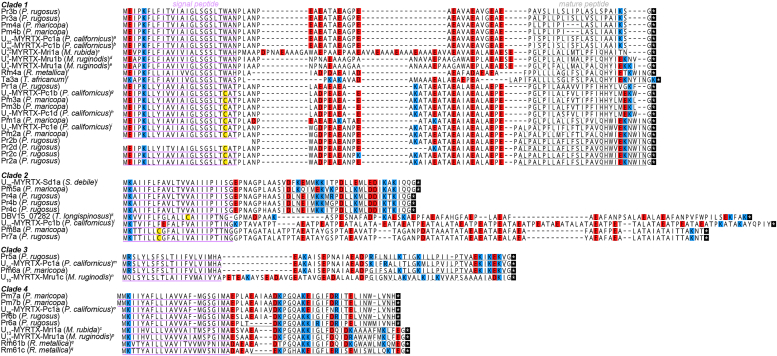
***Pogonomyrmex* venom peptides group into four clades and are homologous with venom peptides of other myrmicine and ectatommine ants.** Signal peptides, confirmed mature peptides and predicted mature peptides are underlined in *purple*, *gray* and *gray* (*dashed*), respectively. Lysine/arginine, aspartate/glutamate, and cysteine residues are highlighted in *blue*, *red*, and *yellow*, respectively. *A*, CAH2461051.1; *B*, CAH2461053.1; *C*, (16); *D*, CAH2461050.1; *E*, CAH2461052.1; *F*, (17); *G*, CAH2461036.1; *H*, CAH2461042.1; *I*, CAH2461044.1; *J*, CAH2461068.1; *K*, TGZ57889.1; *L*, CAH2461081.1; *M*, CAH2461066.1; *N*, CAH2461070.1; *O*, CAH2461071.1; *P*, CAH2461067.1; *Q*, (57).

Analysis of the precursor (prepropeptide) sequences of the *Pogonomyrmex* venom peptides revealed features consistent with other hymenopteran venom peptides of the aculeatoxin gene superfamily, namely a short signal peptide sequence followed by a repetitive and highly anionic propeptide and a cysteine-free mature peptide (Fig. 2) (4). Among these, the precursors encoding Pm1a, Pm2a, Pm3a, Pm3b, Pm4a, and Pm4b from *P. maricopa*, and Pr1a, Pr2a, Pr2b, Pr2c, Pr2d, Pr3a, and Pr3b from *P. rugosus*, constitute a single clade (clade 1); Pm5a, Pm8a, Pr4a, Pr4b, Pr4c, and Pr7a constitute another clade (clade 2); Pm6a and Pr5a, a third (clade 3); and Pm7a, Pr6a, and Pr6b, a fourth (clade 4).

We used the BLASTp algorithm to search the UniProt/GenBank protein database for sequences related to these peptides. For all clades, homologous sequences were detected from other myrmicine and ectatommine ants. Alignments for each clade are shown in Figure 2. For clades 2 and 3, all of the homologous sequences were uncharacterized. In clade 4, U_13_-MYRTX-Mri1a from *Manica rubida* venom, had been tested for insecticidal activity and shown to cause rapid paralysis (median paralytic dose (PD_50_) = 2.9 nmol/g) in blowflies (*Lucilia caesar*), but it was not lethal (16). The *Pogonomyrmex* venom peptides Pm7a/b, Pr6a, and Pr6b have 50, 46, and 52% mature peptide sequence identity with U_13_-MYRTX-Mri1a. For clade 1, three homologous peptides are well characterized: Rm4a, Ta3a, and U_3_-MYRTX-Mri1a, from the venoms of the ants *Rhytidoponera metallica*, *Tetramorium africanum* and *M. rubida*, respectively, modulate mammalian voltage-gated sodium (Na_V_) channels and induce nocifensive behaviors in mice (17). Pm1a, Pm2a, Pr2a/c, and Pr2b/d have 63 to 71% mature peptide sequence identity with Rm4a and Ta3a.

**Figure 3:**
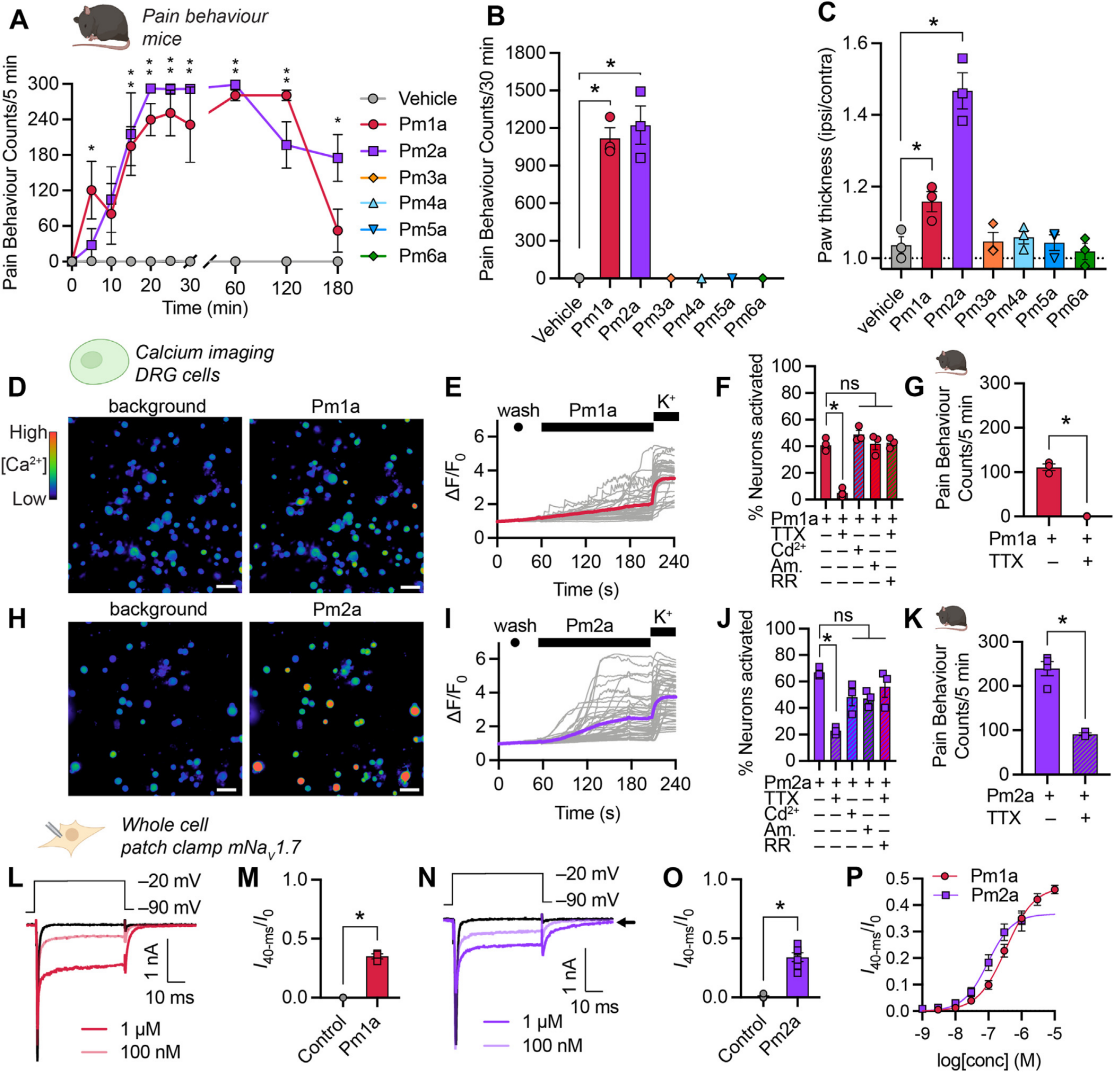
***Pogonomyrmex maricopa* venom peptides, Pm1a and Pm2a, cause long-lasting near maximal spontaneous nocifensive behaviors in mice and modulate neuronal Na**_**V**_**channels.***A*, spontaneous nocifensive behaviors in mice following shallow intraplantar injection of *P. maricopa* venom peptides (20 pmoles; *n* = 3–7 mice per group). ∗*p* < 0.05 (two-way ANOVA with Holm-Šídák’s multiple comparisons to negative control). *B*, cumulative spontaneous pain behaviors in mice 30 min after intraplantar injection. ∗*p* < 0.0001 (one-way ANOVA with Dunnett multiple-comparisons; *n* = 3 mice per group). *C*, paw swelling in mice 1 h after intraplantar injection. ∗*p* = 0.0201 (Pm1a *versus* vehicle), *p* < 0.0001 (Pm2a *versus* vehicle) (one-way ANOVA with Dunnett multiple-comparisons; *n* = 3 mice per group). *D*, representative (of three independent experiments) pseudocolor images illustrating [Ca^2+^]_*i*_ in DRG neurons before (background) and after application of Pm1a (10 nM); the scale bar represents 100 μm. *E*, time course of individual DRG neuron responses to Pm1a. Each trace represents an individual neuron and the *red* trace represents the average response; K^+^, 30 mM KCl (positive control) (representative of four independent experiments). *F*, percentage of DRG neurons activated by Pm1a (10 nM) in the absence or presence of tetrodotoxin (TTX, 1 μM), Cd^2+^ (100 μM), amiloride (Am., 20 μM) or ruthenium *red* (RR, 20 μM). ∗*p* < 0.0001 (one-way ANOVA; *n* = 3–4 independent experiments). *G*, cumulative spontaneous nocifensive behaviors in mice after intraplantar injection of saline or TTX (2 μM) (*n* = 3–4 mice per group), 30 min after injection of Pm1a (20 pmoles).∗*p* < 0.0001 (unpaired *t* test; two-sided). *H*–*K*, equivalent data for Pm2a. *J*, ∗*p* = 0.0004 (one-way ANOVA; *n* = 3–4 independent experiments). *K*, ∗*p* = 0.0005 (unpaired *t* test; two-sided; *n* = 3–4 mice per group). *L*, representative current response from a HEK293 cell expressing mNa_V_1.7 to a step depolarization from −90 to −20 mV in the absence (*black*) and presence of Pm1a (*red*). *M*, sustained current (*I*_40-ms_/peak current (*I*_0_)) before (control) and after the addition of Pm1a (1 μM). ∗*p* = 0.0036 (paired *t* test, two-sided; *n* = 3 cells). *N* and *O*, equivalent data for Pm2a. ∗*p* = 0.0003 (paired *t* test, two-sided; *n* = 5 cells). The *arrow* indicates residual current. *P*, concentration-response relationship for modulation of mNa_V_1.7 where response is [sustained current (*I*_40-ms_)/peak current (*I*_0_)]. Data are expressed as mean ± SEM. DRG, dorsal root ganglion.

Given that *Pogonomyrmex* venoms are dominated by peptides, we hypothesized that one or more of these toxins may be responsible for the characteristic pain from their stings. We chemically synthesized the *P. maricopa* venom peptides Pm1a, Pm2a, Pm3a, Pm4a, Pm5a, and Pm6a and tested their capacity to cause spontaneous nocifensive behaviors in mice following intraplantar injection. Injection (20 pmoles) of either Pm1a or Pm2a but not the other venom peptides caused spontaneous nocifensive behaviors which were gradual in onset and long-lasting (Fig. 3, *A* and *B*). Pm1a and Pm2a also caused swelling of the injected paw (Fig. 3*C*). The time-course and magnitude of effects caused by Pm1a and Pm2a in mice are consistent with *Pogonomyrmex* envenomation symptoms in humans ((1, 8) and personal observations, S. D. R. and J. O. S.). Furthermore, they are distinct from those caused by pore-forming peptides from the venoms of other hymenopterans that are characterized by “short, sharp” envenomation symptoms (3, 4, 5, 6).

Pm1a and Pm2a have 63 to 71% sequence similarity with Rm4a and Ta3a, ant venom peptides that are potent modulators of mammalian Na_V_ channels (17). We therefore hypothesized that Pm1a and Pm2a would have a similar mode-of-action. To investigate the mechanism(s) by which Pm1a and Pm2a cause nociception in mice, we tested their activity on primary cultures of dissociated mouse dorsal root ganglion (DRG) cells, which include sensory neurons responsible for the detection of painful stimuli. Application of Pm1a (10 nM) or Pm2a (10 nM) to mouse DRG cells caused an immediate and sustained increase in fluorescence indicative of increased intracellular Ca^2+^ concentration ([Ca^2+^]_*i*_) in 40.7 ± 2.2% and 67.1 ± 2.0% of neurons, respectively (Fig. 3, *D*–*F*, *H*–*J*). All nonneuronal cells in the cultures were unaffected, indicative of a specific neuronal target(s). When repeated in the presence of 2 μM tetrodotoxin (TTX), a blocker of certain mammalian Na_V_ channels (Na_V_1.1, Na_V_1.2, Na_V_1.3, Na_V_1.4, Na_V_1.6, and Na_V_1.7) the percentage of neurons activated by Pm1a and Pm2a was reduced to 5.0 ± 1.4% (*p* < 0.0001, unpaired *t* test) and 22.7 ± 1.6% (*p* < 0.0001, one-way ANOVA with Dunnett multiple comparison test), respectively (Fig. 3, *F* and *J*). By contrast, neither Cd^2+^ (100 μM, a nonselective inhibitor of voltage-gated calcium channels), amiloride (20 μM, a nonselective inhibitor of acid-sensing ion channels) nor ruthenium red (20 μM, a nonselective antagonist of transient receptor potential cation channels and ryanodine receptors) altered the percentage of neurons activated by Pm1a or Pm2a. Pm1a- and Pm2a-induced spontaneous nocifensive behaviors in mice were completely (Pm1a; *p* < 0.0001, unpaired *t* test) and partially (Pm2a; *p* = 0.0005, unpaired *t* test) ameliorated by injection of 2 μM TTX (Fig. 3, *G* and *K*). Together, these data suggest that the effects of these peptides on both DRG neurons and *in vivo* are primarily mediated by TTX-sensitive Na_V_ channels, and for Pm2a, additionally by one or more TTX-resistant ion channel(s)—likely Na_V_1.8 and Na_V_1.9 (see data below).

Na_V_ channels are essential for action potential generation and propagation in neurons. Mammalian sensory neurons express at least six of the nine known Na_V_ isoforms, including Na_V_1.6, Na_V_1.7 (both TTX-sensitive), Na_V_1.8 and Na_V_1.9 (both TTX-resistant), for which gain-of-function mutations have been linked with pain (18, 19, 20, 21). Using whole-cell voltage-clamp electrophysiology, we investigated the effects of Pm1a and Pm2a on mouse Na_V_1.7. Representative mNa_V_1.7 current responses to a step depolarization of the cell membrane voltage from −90 to −20 mV in the absence and presence of Pm1a and Pm2a are shown in Figure 3, *L* and *N*, respectively. Consistent with the results of our calcium imaging experiments using mouse DRGs, both Pm1a and Pm2a are potent modulators of mNa_V_1.7. Pm1a reduced peak current amplitude, caused a sustained current (at 1 μM: 0.35 ± 0.02 fraction of peak; *p* = 0.0036, paired *t* test) with an EC_50_ of 352 ± 58 nM, and induced a tail current upon repolarization (Fig. 3, *L*, *M*, and *P*). Similarly, Pm2a reduced peak current amplitude, caused a sustained current (at 1 μM: 0.34 ± 0.04 fraction of peak; *p* = 0.0003, paired *t* test) with an EC_50_ of 102 ± 18 nM, and induced a tail current upon repolarization that did not completely inactivate (Fig. 3, *N*–*P*).

**Figure 4:**
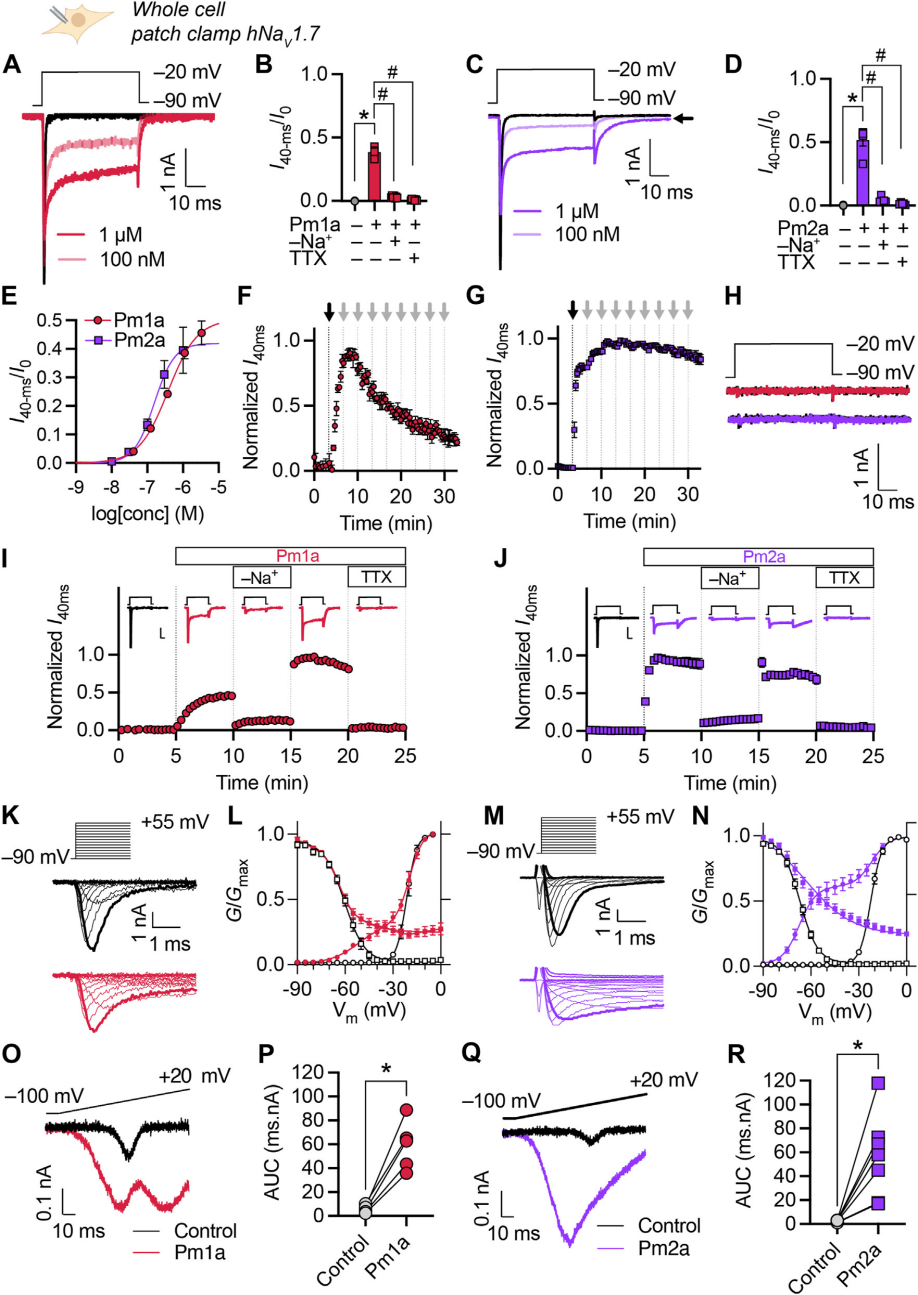
***Pogonomyrmex maricopa* venom peptides, Pm1a and Pm2a, are potent modulators of human Na**_**V**_**1.7.***A*, representative current response from a HEK293 cell expressing hNa_V_1.7 to a step depolarization from −90 to −20 mV in the absence (*black*) and presence of Pm1a (*red*). *B*, sustained current (*I*_40-ms_/peak current (*I*_0_)) before (control) and after the addition of Pm1a (1 μM), in Na^+^-free buffer or with TTX (1 μM). ∗*p* = 0.0003 (paired *t* test, two-sided; *n* = 4 cells); ^#^*p* < 0.0001 (unpaired *t* test, two-sided; *n* = 7 cells). *C* and *D*, equivalent data for Pm2a. ∗*p* = 0.0004 (paired *t* test, two-sided; *n* = 5 cells); ^#^, *p* < 0.0001 (unpaired *t* test, two-sided; *n* = 5 cells). Data for Pm2a, presented here and in subsequent panels, were analyzed and are shown without leak subtraction. The *arrow* indicates residual current. *E*, concentration-response relationship for modulation of hNa_V_1.7 where response is [sustained current (*I*_40-ms_)/peak current (*I*_0_)]. *F*, washout protocol measuring Pm1a-induced hNa_V_1.7 persistent current over time (*n* = 4 cells). The addition of Pm1a (1 μM) and washouts (extracellular solution) are indicated by *black* and *gray arrows*, respectively. *G*, equivalent data for Pm2a (1 μM; *n* = 4 cells). *H*, representative (of seven cells (Pm1a) and six cells (Pm2a)) current responses from HEK293 cells to a step depolarization from −90 to −20 mV in the absence (*black*) and presence of Pm1a (1 μM; *red*) or Pm2a (1 μM; *purple*). *I*, effects of Na^+^-free buffer and TTX (1 μM) on Pm1a-induced hNa_V_1.7 persistent current over time (*n* = 7 cells). *Inset*: representative current responses, at the end of each step, to a step depolarization from −90 to −20 mV. The scale bar represents 1 nA, 10 ms. *J*, equivalent data for Pm2a (1 μM; *n* = 5 cells). *K*, representative *I-V* traces for hNa_V_1.7 channels expressed in HEK293 cells before (*top*) and after addition of Pm1a (3 μM, *bottom*). Traces corresponding to −20 mV steps are bold. *L*, hNa_V_1.7 *G-V* (*circles*) and SSFI (*squares*) curves, before (*white*) and after addition of Pm1a (3 μM, *red*; *n* = 3 cells). *M* and *N*, equivalent data for Pm2a (1 μM; *n* = 9 cells). *O*, representative current response from a HEK293 cell expressing hNa_V_1.7 to a voltage ramp from −100 to +20 mV (1 mV/ms) before (*black*) and after addition of Pm1a (3 μM, *red*). *P*, current response [AUC (ms.nA)] to voltage ramp before and after addition of Pm1a (3 μM). ∗*p* = 0.0030, paired *t* test; *n* = 5 cells. *Q* and *R*, equivalent data for Pm2a (1 μM, *purple*). ∗*p* = 0.0059, paired *t* test; *n* = 7. Data are expressed as mean ± SEM. AUC, area under the curve; SSFI, steady-state fast inactivation; TTX, tetrodotoxin.

We repeated these experiments using human Na_V_1.7 channels and observed similar effects: Pm1a caused a sustained current (at 1 μM: 0.39 ± 0.02 of peak; *p* = 0.0003, paired *t* test *versus* control) with an EC_50_ of 403 ± 96 nM (Fig. 4, *A*, *B* and *E*) and induced a tail current upon repolarization. Pm2a caused a sustained current (at 1 μM: 0.52 ± 0.05 of peak; *p* = 0.0004, paired *t* test), with an EC_50_ of 154 ± 17 nM, and induced a tail current upon repolarization which did not completely inactivate (Fig. 4, *C*–*E*). Subsequent experiments were performed with human Na_V_ channels.

Pm1a (1 μM)-induced hNa_V_1.7 persistent current was slowly reversible with repeated wash steps over 30 min, while Pm2a (1 μM)-induced hNa_V_1.7 persistent current was near-irreversible (Fig. 4, *F* and *G*). The observed effects on hNa_V_1.7 current by Pm1a and Pm2a were entirely mediated *via* the Na_V_ channel itself (as opposed to another channel or *via* direct pore-formation by the peptides) as evidenced by the absence of inward current upon addition of Pm1a (1 μM) or Pm2a (1 μM) to nontransfected HEK293 cells (Fig. 4*H*) and the absence of inward current in hNa_V_1.7-expressing cells in Na^+^-free buffer or in the presence of TTX (1 μM) (Fig. 4, *B*, *D*, *I*, and *J*).

In current-voltage (*I*-V) experiments at hNa_V_1.7, both Pm1a and Pm2a caused a large hyperpolarizing shift in the voltage-dependence of channel activation (Pm1a 3 μM: ΔV_50_ = −31.4 ± 1.6 mV, *p* = 0.0025, paired *t* test; Pm2a 1 μM: ΔV_50_ = −44.0 ± 0.5 mV, *p* < 0.0001, paired *t* test) (Fig. 4, *K*–*N*) which fitted to a double-Boltzmann distribution (the second component of which exhibited no difference in voltage-dependence of channel activation to the negative controls (Pm1a 3 μM: *p* = 0.9102, paired *t* test; Pm2a 1 μM: *p* = 0.4228, paired *t* test)). No change in voltage-dependence of steady-state fast inactivation (SSFI) was observed (Pm1a 3 μM: ΔV_50_ = −3.7 ± 1.4 mV, *p* = 0.0774, paired *t* test; Pm2a 1 μM: ΔV_50_ = 4.3 ± 2.0 mV, *p* = 0.0623, paired *t* test), although at depolarizing potentials a large proportion of channels remained available for activation. Together these effects on channel activation and inactivation result in a substantial window current which spans the resting membrane potential for neurons (∼−70 mV).

Na_V_1.7 is important for generating ramp currents that amplify small depolarizations and bring the membrane potential to the threshold needed to fire action potentials. We therefore assessed the effects of Pm1a and Pm2a had on hNa_V_1.7 ramp currents. In HEK293 cells expressing hNa_V_1.7, depolarizing voltage ramps from −100 to +20 mV (1 mV/ms) caused a small ramp current, which in the presence of Pm1a (3 μM) increased from 5.6 ± 1.4 ms.nA to 59.2 ± 9.3 ms.nA (*p* = 0.0030, paired *t* test; Fig. 4, *O* and *P*); and in the presence of Pm2a (1 μM), the area under the curve increased from 2.4 ± 0.3 ms.nA to 56.5 ± 13.2 ms.nA (*p* = 0.0059, paired *t* test; Fig. 4, *Q* and *R*). The two-component response observed with Pm1a is consistent with previous studies of ramp currents at Na_V_1.3 and Na_V_1.6, where two-component ramp currents are observed—the first peak is due to channel activation followed by inactivation, and the second peak is related to sustained current (22, 23).

**Figure 5:**
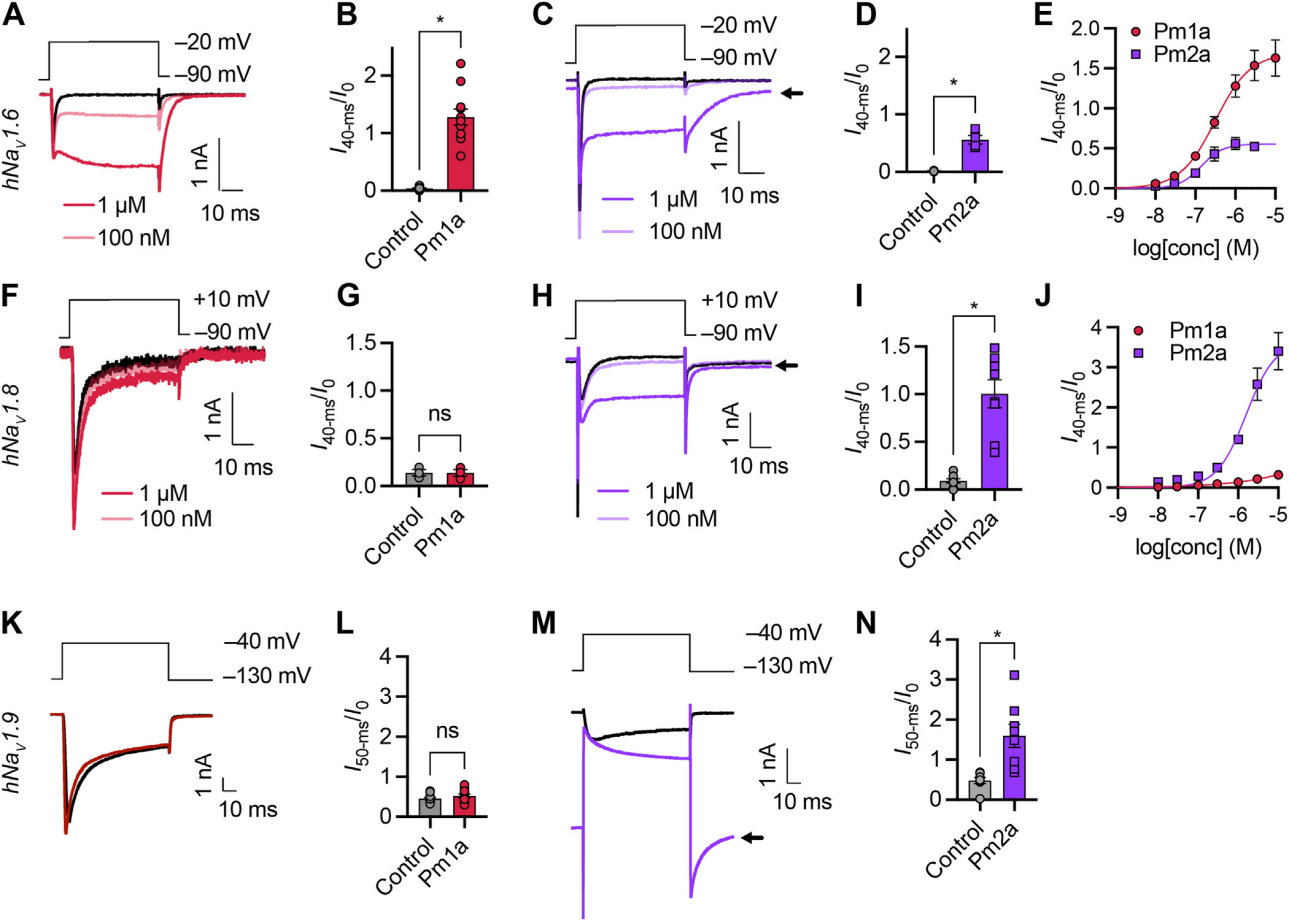
**Modulation of other Na**_**V**_**channel subtypes by Pm1a and Pm2a.***A*, representative current response from a HEK293 cell expressing hNa_V_1.6 to a step depolarization from −90 to −20 mV in the absence (*black*) and presence of Pm1a *(red*). *B*, sustained current (*I*_40-ms_/peak current (*I*_0_)) before (control) and after the addition of Pm1a (1 μM). ∗*p* < 0.0001 (paired *t* test, two-sided; *n* = 11 cells). *C* and *D*, equivalent data for Pm2a. ∗*p* = 0.0048 (paired *t* test, two-sided; *n* = 4 cells). Data for Pm2a, presented here and in subsequent panels, were analyzed and are shown without leak subtraction. The *arrow* indicates residual current. *E*, concentration-response relationships for modulation of hNa_V_1.6 where response is [sustained current (*I*_40-ms_)/peak current (*I*_0_)]. *F*, representative current response from a CHO cell expressing hNa_V_1.8 to a step depolarization from −90 to +10 mV in the absence (*black*) and presence of Pm1a (*red*). *G*, sustained current (*I*_40-ms_/peak current (*I*_0_)) before (control) and after the addition of Pm1a (1 μM). n.s., *p* = 0.9756 (paired *t* test, two-sided; *n* = 3 cells). *H* and *I*, equivalent data for Pm2a. ∗, *p* = 0.0002 (paired *t* test, two-sided; *n* = 8 cells). *J*, concentration-response relationship for modulation of hNa_V_1.8 where response is [sustained current (*I*_40-ms_)/peak current (*I*_0_)]. *K*, representative current response from a HEK293 cell expressing hNa_V_1.9 to a step depolarization from −130 to −40 mV in the absence (*black*) and presence of Pm1a (1 μM; *red*). *L*, sustained current (*I*_40-ms_/peak current (*I*_0_)) before (control) and after the addition of Pm1a (1 μM). n.s., *p* = 0.0525 (paired *t* test, two-sided; *n* = 11 cells). *M* and *N*, equivalent data for Pm2a. ∗*p* = 0.0052 (paired *t* test, two-sided; *n* = 8 cells). CHO, Chinese hamster ovary.

At human Na_V_1.6, Pm1a caused a sustained current (at 1 μM: 1.28 ± 0.14 of buffer peak; *p* < 0.0001, paired *t* test *versus* control) with an EC_50_ of 285 ± 47 nM and induced a tail current upon repolarization (Fig. 5, *A*, *B*, and *E*). Pm2a caused a sustained current (at 1 μM: 0.56 ± 0.07 of buffer peak; *p* = 0.0048, paired *t* test), with an EC_50_ of 176 ± 56 nM, and induced a tail current upon repolarization which did not completely inactivate (Fig. 5, *C*–*E*).

At human Na_V_1.8, Pm1a had no effect on persistent current at the highest concentrations tested (Fig. 5, *F*, *G*, and *J*). An EC_50_ could not be calculated. By contrast, Pm2a caused a large sustained current (at 1 μM: 1.00 ± 0.14 of peak; *p* = 0.0002, paired *t* test), with an EC_50_ of 2.6 ± 0.7 μM, as well as a large tail current upon repolarization that did not completely inactivate (Fig. 5, *H* and *I*).

At hNa_V_1.9, at a concentration of 1 μM, Pm1a had no effect (*p* = 0.0525, paired *t* test; Fig. 5, *K* and *L*) while Pm2a caused a large sustained current (at 1 μM: 1.60 ± 0.29 of peak; *p* = 0.0052, paired *t* test) and a large tail current that did not inactivate on repolarization to −130 mV resulting in a large ”leak” current (Fig. 5, *M* and *N*). At the same concentration, Pm2a did not produce leak currents in nontransfected HEK293 cells (Fig. 4*H*), suggesting that this inward current is being mediated *via* the Na_V_1.9 channel itself, and not due to a loss in the patch-clamp seal due to disruption of the membrane. The data suggest that hNa_V_1.9 may be more sensitive than hNa_V_1.8 to Pm2a; and that the EC_50_ of Pm2a at Na_V_1.9 is likely to be <1 μM. Together these data indicate that Pm1a is selective for TTX-sensitive over TTX-resistant Na_V_ subtypes, while Pm2a is less so (Table 2). These results may explain our observations of TTX-resistant activity for Pm2a in primary cultures of mouse DRG neurons and *in vivo* (Fig. 3, *J* and *K*).

**Table 2:** Potency (nM) of *Pogonomyrmex maricopa* Na_V_ channel toxins at Na_V_ channel subtypes

Peptide	Primary structure	hNa_V_1.6	mNa_V_1.7	hNa_V_1.7	hNa_V_1.8	hNa_V_1.9
Pm1a	GLPLLALLFSLPVLQHWIEKNWIN^a^	285 ± 47	352 ± 58	403 ± 96	>10,000	>1000
Pm2a	ALPALPLLMFLFTLPALQHWIEKNWIN^a^	176 ± 56	102 ± 18	154 ± 17	2600 ± 700	<1000

a *C*-terminal amidation.

Other activities reported of *Pogonomyrmex* venoms include >95% lysis of mouse erythrocytes (4 μg/ml venom) and at doses of 50 to 100 mg/kg, toxicity to insect larvae (9, 24). We tested whether any of the six synthetic *P. maricopa* venom peptides were hemolytic or cytotoxic, or paralytic to insects. Only Pm6a caused lysis of human erythrocytes with a median hemolytic concentration (HC_50_) of 618 ± 148 nM; it was also cytotoxic to HEK293 cells with a median cytotoxic concentration (CC_50_) of 465 ± 359 nM.

**Figure 6:**
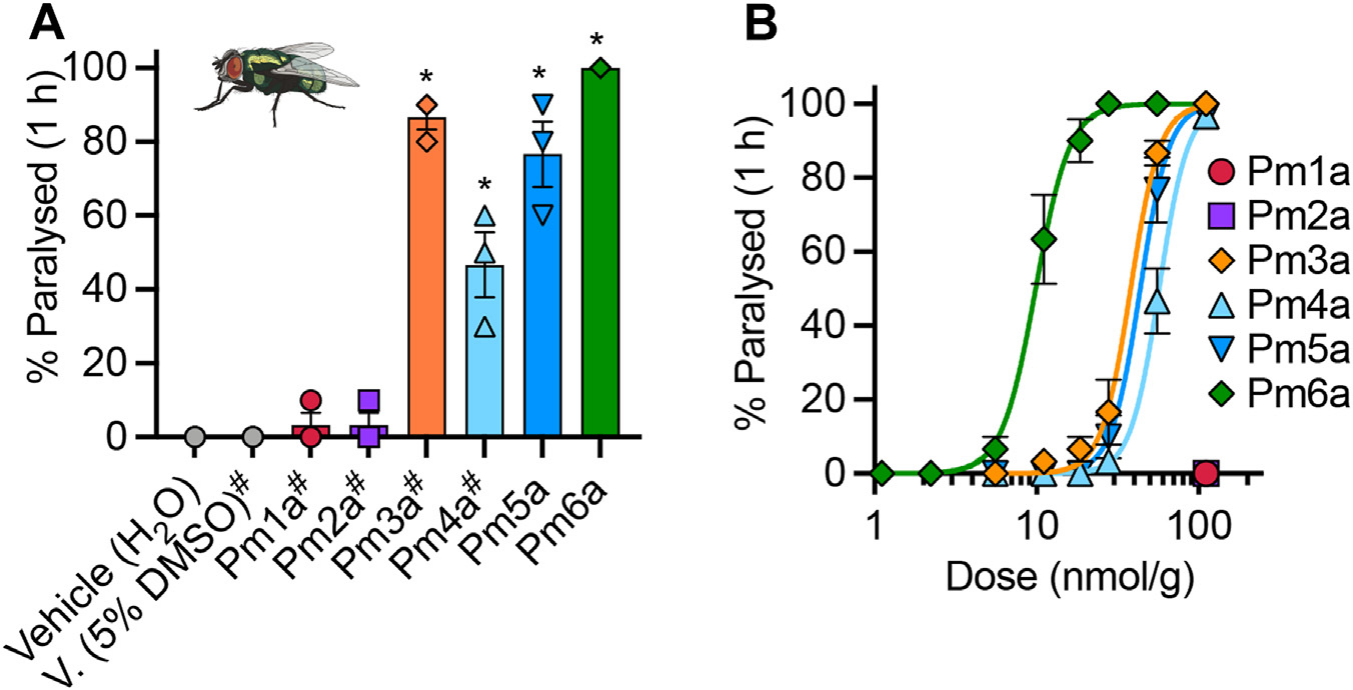
**PM1a and Pm2a are vertebrate-specific.***A*, percentage of flies paralyzed 1 h after intrathoracic injection of each peptide (107 nmol/g; *n* = 3 independent experiments). ^#^, 5% DMSO. ∗*p* = 0.9614 (Pm1a), *p* = 0.9614 (Pm2a), *p* < 0.0001 (Pm3a), *p* = 0.0001 (Pm4a), *p* < 0.0001 (Pm5a), *p* < 0.0001 (Pm6a), one-way ANOVA with Dunnett multiple-comparisons to negative controls. *B*, dose-response relationships of peptides. Data are expressed as mean ± SEM and (*panel B*) fitted with a nonlinear regression with variable slope (four parameters). DMSO, dimethyl sulfoxide.

Next, we tested whether any of the synthetic *P. maricopa* venom peptides could paralyze insects. Intrathoracic injection of Pm3a in blowflies (*L. caesar*) caused a slowly developing temporary paralysis (PD_50_[1 h] = 38.1 ± 1.6 nmol/g; Fig. 6, *A* and *B*). Pm4a, Pm5a and Pm6a caused rapid paralysis (PD_50_[1 h] = 57.0 ± 1.7 nmol/g, 43.7 ± 1.9 nmol/g and 9.9 ± 0.4 nmol/g; Fig. 6, *A* and *B*), which for Pm5a and Pm6a was irreversible (flies checked at 24 h). By contrast, neither Pm1a nor Pm2a (up to a dose of 111 nmol/g (2 nmol)) had an effect on blow flies (Fig. 6, *A* and *B*). These data suggest that peptides related to one or all of Pm3a, Pm4a, Pm5a, and Pm6a underlie the insecticidal activity observed for *Pogonomyrmex badius* venom (9). Furthermore, they are consistent with the hypothesis that Pm1a and Pm2a have evolved for defense against vertebrates.

## Discussion

*Pogonomyrmex* ants are notorious for their painful stings. This has led to several attempts to study their venoms and uncover the active agent(s). Most previous studies on *Pogonomyrmex* venoms focussed on protein composition and enzymatic activity (9, 12, 25). Here, we show that while proteins/enzymes are present in *Pogonomyrmex* venoms, they are a minor component relative to peptides. Furthermore, consistent with an analysis of *P. barbatus* venom by Bernheimer *et al.* (24), our data reveal that peptides are the major active components of *Pogonomyrmex* venoms.

We demonstrate here that the venom composition of two *Pogonomyrmex* species, *P. maricopa* and *P. rugosus*, is similar. *Pogonomyrmex californicus* venom also appears to contain peptides that are homologous to those detected in *P. maricopa* and *P. rugosus* (26). Further research will be necessary to establish whether more distantly related *Pogonomyrmex* species have similar venoms, although several lines of evidence suggest that this is likely to be the case—of the *Pogonomyrmex* species tested, all produce similar symptoms in envenomated humans ((7) and author observations, S. D.R. and J. O. S.); diverse harvester ant venoms exhibit similar potency of lethality in mammals (7); and diverse harvester ant venoms exhibit cross-allergenicity (7, 14, 15). Together, these reports paint a picture of highly similar venom composition across the genus *Pogonomyrmex*.

We show that some of the peptides in *P. maricopa* venom have activity in insects—although, with the exception of Pm6a, they have low potency. *Pogonomyrmex* are thought to use their venom exclusively for defense, although other arthropods are collected as a food source (27). Whether *Pogonomyrmex* use their sting to incapacitate or kill arthropod prey, as is the case for some other ants, is not yet known, but the presence of one or more invertebrate-active venom components suggests that this may be possible. Alternatively, it is possible that this activity may reflect a function for defense against invertebrate predators or competitors (28), although there is little evidence of major predation pressure by any invertebrates on *Pogonomyrmex* (29). Finally, it remains possible that *Pogonomyrmex* venoms have evolved primarily for defensive use and the observed invertebrate activity is a side-effect of a defensive the mode of action.

Pm6a, which was active in invertebrates, was also hemolytic and cytotoxic to human erythrocytes and HEK293 cells, respectively. Homologues of Pm6a are present in other *Pogonomyrmex* venoms (Fig. 2) and it seems likely that the previously reported hemolytic effects of *P. badius* venom are due to such a homologue(s) (9). Similarly the hemolytic polypeptide “barbatolysin” identified in the venom of *P. barbatus* (24) may also be related to Pm6a—indeed, the estimated mass of barbatolysin (∼3000–3500 Da) is consistent with that of Pm6a (3117 Da). While Pm6a did not cause nociception in mice at the doses tested here, the observed toxicity of this peptide may nonetheless reflect a defensive role.

The remaining venom peptides are vertebrate-selective and cause long-lasting nocifensive behaviors in mice, likely *via* potent modulation of sensory neuron Na_V_ channels, thereby explaining the painful envenomation symptoms in humans. This family of toxin is shared by several species outside of the genus *Pogonomyrmex*, including another myrmicine ant *T. africanum* and the ectatommine ant, *R. metallica*, where they serve the same function, as vertebrate-selective defensive toxins (17). The limited divergence of these peptides (Fig. 2) between these different lineages of formicoid ants suggests that a near identical vertebrate-specific Na_V_ channel toxin(s) was already present in the venom of their common ancestor, where it likely served a similar function. As the formicoid ants diverged, some lineages lost this toxin family (17) (in some cases venom was lost entirely), while others retained it, and in the case of *Pogonomyrmex*, it is now the dominant part of the venom.

Two questions arise from these observations: First, what was the selection pressure(s) that first drove the emergence of this toxin family in the early Formicoid ants? The common ancestor of the ectatommine and myrmicine ants is predicted to have inhabited the Neotropical realm during the Cretaceous period approximately 90 Mya (30). The inferred existence of a vertebrate-selective defensive toxin in the venom of this ant points to strong vertebrate selection pressure at this time; notably, it coincides with the radiation of the Alvarezsoroids, small therapod dinosaurs, which exhibit anatomical features suggestive of obligate myrmecophagy (31). Alternatively, putative myrmecophagous mammals have been described from the Jurassic (32), and it is possible that descendants of these could have maintained this ecological niche into the Cretaceous. Further additions to the fossil record will be of interest in retracing the predator-prey interactions of the early ants.

Second, what selection pressure(s) has since contributed to the maintenance of this toxin family in modern *Pogonomyrmex*? An obvious candidate is predation by horned lizards of the genus *Phrynosoma*. Horned lizards are primarily myrmecophageous (33) and some species prey almost exclusively on *Pogonomyrmex*—studies of *Phrynosoma cornutum* report diets comprising 89 to 98% *Pogonomyrmex* spp. (29, 34); Predation by horned lizards can have a major impact on harvester ant populations (29)—one study reports the predation by *P. cornutum* of 213 individual *Pogonomyrmex* ants in a single day, and calculated that at the study site, the horned lizard population was utilizing *Pogonomyrmex* spp. at or close to their maximum exploitation level (29); Horned lizards exhibit multiple behavioral, anatomical and physiological traits that appear to represent specialized adaptations to predation of *Pogonomyrmex* ants. These include specializations in foraging strategy (29), prey capture and handling (35) and digestive tract anatomy and physiology (36), as well as reduced sensitivity to *Pogonomyrmex* venom toxicity (37). These observations have led to suggestions that *Pogonomyrmex* and horned lizards may be in a co-evolutionary arms race, and that the potency of *Pogonomyrmex* venom on vertebrates may reflect this. However, as previously noted (7), there are some inconsistencies with this hypothesis: early *Pogonomyrmex* are thought to have evolved in what is now northern South America (27) in the absence of horned lizards or any known ancestor of horned lizards. Only after spreading to North America did their range overlap with that of *Phrynosoma*; *Pogonomyrmex* species that have evolved in South America, and have never encountered horned lizards or the ancestors during their evolution, have similarly potent (7) and painful (1) venoms. A second candidate for the selection pressure underlying the maintenance of this toxin family in modern *Pogonomyrmex* is competition with granivorous vertebrates *e.g.* kangaroo rats (*Dipodomys* spp.) (7, 27). *Pogonomyrmex* spp. store harvested seeds in chambers within the nest known as “granaries”. Caches as large as >500,000 seeds (>500 g) and 58,000 seeds have been reported in nests of *P. badius* and *Pogonomyrmex occidentalis*, respectively (38, 39). It is easy to imagine the temptation that such seed caches might represent for other granivores, and that it might therefore be highly advantageous for *Pogonomyrmex* to be able to defend against pilfering. However, an inconsistency with this hypothesis lies in the fact that certain species of *Pogonomyrmex e.g. Pogonomyrmex montanus*, do not store seeds (40), yet retain an equally efficacious defensive sting (7). Further studies of present-day and historical *Pogonomyrmex*-predator interactions will be valuable in addressing these questions.

The effects of *Pogonomyrmex* stings did not go unnoticed by the early human inhabitants of the Americas (11). For several people groups inhabiting Central and Southern present-day California, stings of *P. californicus* were used in various medical applications and initiation rituals (11, 41). The latter sometimes involved the ingestion, on a fasted stomach, of up to hundreds of live ants. Initiates of this practice reported internal pain, presumably resulting from mass envenomation by the swallowed ants, followed by loss of consciousness and hallucinations. These reports led to suggestions that *Pogonomyrmex* venoms may contain one or more psychoactive components. Here, we have demonstrated the presence of potent Na_V_ channel toxins in *Pogonomyrmex* venoms. Given the key role of Na_V_ channels in the human central nervous system (CNS), it is reasonable to expect that even trace amount of such toxins could have a profound impact on CNS function. However, the *Pogonomyrmex* venom Na_V_ channel toxins are peptidic, and it is widely accepted that most peptides are unable to access the CNS when delivered in the periphery. Further studies would be required to determine if, as a result of a mass envenomation event, trace amounts of the *Pogonomyrmex* venom Na_V_ channel toxins are able to access the CNS and have a direct action on CNS function or whether the reported symptoms are due to an indirect action of the venom on the CNS or another factor(s).

The *Pogonomyrmex* Na_V_ channel toxins, Pm1a and Pm2a, together with the previously described Rm4a, Ta3a, and Pc1a (17), and likely other orthologues from other ant species, represent a new class of Na_V_ channel modulator. They are structurally and functionally unrelated to any previously described class of peptidic Na_V_ modulator and instead their complex effects on Na_V_ channel currents more closely resemble those of the “site 2” alkaloid toxins *e.g.* batrachotoxin (found in poison frogs of the genus *Phyllobates* and poison birds of the genera *Pitohui* and *Ifrita*) (42, 43).

Na_V_ channel site 2 primarily comprises residues of the S6 helices of each channel domain which together line the hydrophobic central cavity of the channel pore (44). Binding of site 2 toxins causes (i) a characteristic large shift of the voltage-dependence of activation toward more negative potentials; (ii) slowing or inhibition of fast inactivation; and (iii) a reduction in Na^+^ conductance and selectivity (45, 46). Furthermore, the double-Boltzmann distribution of the voltage-dependence of activation observed for the ant venom Na_V_ channel toxins is also shared by certain site two toxins (42, 43, 47), which in each case was interpreted as reflective of two separate populations of channels: toxin-modified and nonmodified.

The similarities between the effects of the ant venom Na_V_ channel toxins and those of the established site 2 toxins could be interpreted as overlapping binding sites. However, how these peptide toxins could access the inner pore of the channel remains to be established. Alternatively, it is possible that the complex effects on channel gating results from binding at other sites such as sites 3 and 4 of the voltage-sensing domains. Finally, it remains possible that the ant venom Na_V_ channel toxins may interact with the channel at a site(s) that is yet-to-be defined. Further structural and functional studies of toxin-channel interactions will be necessary to determine the channel binding site(s) of the ant venom Na_V_ channel toxins and how binding translates into the observed effects on channel gating.

Among the ant venom Na_V_ channel toxins described so far, there are some differences in efficacy, potency, and selectivity at human Na_V_ channel subtypes. For example, here, we show that while Pm1a is selective for TTX-sensitive Na_V_ channel subtypes, this is not the case for Pm2a (Table 2). More detailed structure-activity relationship studies of these peptides will shed light on the structural features underlying these differences. There is the potential that these peptides, or derivatives thereof, may find utility as tools to investigate unique aspects of mammalian Na_V_ channel structure and function.

## Experimental procedures

### Venom and venom apparatus collection

Approximately 40 adult female worker caste *P. maricopa* were collected from a single colony entrance in Cochise County, Arizona. The venom apparatus were dissected in PBS. Those used for RNA-seq (approximately 20), were placed directly in RNAlater and stored at −20 °C. For venom collection, venom was squeezed from the dissected venom reservoirs and venom ducts of ∼20 individuals into 20 μl water and stored at −20 °C. The total amount of venom was estimated from *A*_280_ measured using a Nanodrop spectrophotometer (Thermo Fisher Scientific).

Approximately 30 adult female worker caste *P. rugosus* were collected from a single colony entrance in Pima County, Arizona. Venom apparatus and venom were collected and stored as for *P. maricopa*.

### Transcriptome sequencing and assembly

Total RNA was extracted from the venom apparatus of *P. maricopa* and *P. rugosus* using TRIzol (Life Technologies). Complementary DNA library preparation and sequencing was performed by the Institute for Molecular Bioscience Sequencing Facility, the University of Queensland. Dual-indexed libraries were constructed with the TruSeq-3 Stranded mRNA Sample Prep Kit (Illumina) with oligo (dT) selection and an average insert size of 180 base pairs. The samples were pooled in a batch of 21 samples, and 150-cycle paired-end sequencing was performed on an Illumina NextSeq 500 instrument. Adapter trimming of demultiplexed raw reads was performed using fqtrim v0.9.7 (https://github.com/gpertea/fqtrim) (48) followed by quality trimming and filtering using prinseq-lite v0.20.4 (49). Error correction was performed using BBnorm tadpole, part of the BBtools package. Trimmed and error-corrected reads were assembled using Trinity v2.4.0 (50) with a k-mer length of 31 and a minimum k-mer coverage of 2. Assembled transcripts were annotated using a BLASTX (51) search (E value setting of 1e^−3^) against the UniRef90 database. Estimates of transcript abundance were performed using the RSEM (https://github.com/trinityrnaseq/trinityrnaseq/wiki/Trinity-Transcript-Quantification) (52) plugin of Trinity (align_and_estimate_abundance). Using TransDecoder, transcripts were translated and filtered to open-reading frames (>30 amino acid residues). These were used as search databases for ProteinPilot.

### Mass spectrometry

A combination of top-down proteomics of native and reduced and alkylated venom, and bottom-up proteomics of reduced, alkylated and trypsin-digested venom was used to examine the polypeptide composition of our *P. maricopa* and *P. rugosus* venom samples. Aliquots of venom (10 μg each) were dried by vacuum centrifugation. Gas phase reduction and alkylation was performed according to the protocol described by Hale *et al.* (53) 100 μl of reduction/alkylation reagent (50% (v/v) ammonium carbonate, 48.75% acetonitrile (ACN), 1% 2-iodoethanol, 0.25% triethylphosphine) was added to the lid of each 1.5 ml tube containing dried venom, which was then inverted, closed, and incubated at 37 °C for 90 min. One aliquot of reduced and alkylated venom was then digested by incubating with trypsin (20 ng/μl) overnight at 37 °C, according to the manufacturer’s instructions (Sigma-Aldrich).

Three venom samples for each species (10 μg each)—native venom, reduced and alkylated venom, and reduced, alkylated and trypsin-digested venom—were analyzed using liquid chromatography-tandem MS . Samples were separated on a Nexera UHPLC (Shimadzu) with a Zorbax stable-bond C18 column (2.1 × 100 mm; particle size, 1.8 μm; pore size, 300 Å; Agilent Technologies), using a flow rate of 180 μl/min and a gradient of 1 to 40% solvent B (90% ACN and 0.1% formic acid) in 0.1% formic acid over 25 min, 40 to 80% solvent B over 4 min, and analyzed on an AB Sciex 5600 TripleTOF (SCIEX; operated with Analyst TTF v1.8) mass spectrometer equipped with a Turbo-V source heated to 550 °C. MS survey scans were acquired at 300 to 1800 mass/charge ratio (m/z) over 250-ms, and the 20 most intense ions with a charge of +2 to +5 and an intensity of at least 120 counts were selected for MS/MS. The unit mass precursor ion inclusion window mass within 0.7 Da and isotopes within 2 Da were excluded from MS/MS, with scans acquired at 80 to 1400 m/z over 100-ms and optimized for high resolution. Using ProteinPilot v5.0 (SCIEX), MS/MS spectra were searched against the translated venom-apparatus transcriptome (MS and MS/MS tolerance of 0.05 and 0.1 Da, respectively). False discovery rate analyses were generated by ProteinPilot default method which uses a decoy database.

Transcripts encoding venom components were then manually examined using the Map-to-Reference tool of Geneious v10.2.6 (https://www.geneious.com/) (54), where “masked” homologues were extricated from assembled transcripts and contaminant transcripts (derived from multiplexing) and erroneous transcripts (derived from misassembly) were discarded. This information was then reincorporated back into each complete transcriptome, estimation of transcript abundance repeated, and a second, final ProteinPilot search performed. Peptides identified by ProteinPilot were validated by comparison of experimentally derived MS/MS peaks against a theoretical peak list generated using MS-Product in ProteinProspector v5.22.1 (http://prospector.ucsf.edu/prospector/cgi-bin/msform.cgi?form=msproduct).

### Peptide synthesis

Peptides were produced using Fmoc solid-phase peptide synthesis at 0.1 mmol scale. Protecting groups used were Lys/Trp/His(Boc), Ser/Thr/Tyr(tBu), Asp/Glu(OtBu), Asn/Gln/His(Trt), and Arg(Pbf). Peptides were assembled on Rink-amide ProTide resin (CEM, Matthews, NC) on a CEM Liberty Prime HT24 microwave synthesizer (CEM Corp) using *N*,*N*′-diisopropylcarbodiimide (DIC)/oxyma and Fmoc groups were removed with 20% pyrrolidine, as per manufacturer’s protocols. Peptides were released from resin by treatment with 95% TFA/2.5% H_2_O/2.5% triisopropyl silane. Peptides were precipitated with 15 ml ice-cold ether, extracted in A/B 50/50 (A: 0.05% TFA, B: 90% ACN, and 0.045% TFA) and lyophilized prior to purification. Peptides were purified on a Shimadzu Prominence LC-20AT RP-HPLC system equipped with a SPD-20AV UV detector and a FRC-10A fraction collector using a Agilent Zorbax 300SB-C18 column (150 × 21.2 mm; particle size, 5 μm) using a flow rate of 16 ml/min and a gradient of 30 to 70% solvent B over 40 min. Fractions of interest were lyophilized and purity assessed using electrospray ionization MS and analytical RP-HPLC. Pure fractions of each peptide were lyophilized, pooled, and stored at room temperature until use. Stock solutions of Pm5a and Pm6a were prepared by dissolving each lyophilized peptide in 100% H_2_O (1 mM final concentration). Stock solutions of Pm1a, Pm2a, Pm3a, and Pm4a were prepared by dissolving each lyophilized peptide first in 100% dimethyl sulfoxide (DMSO) then diluting to 1 mM peptide, 5% DMSO (v/v) in H_2_O.

### Pain behavior experiments

Male 5 to 8 week old C57BL/6J mice used for behavioral experiments were purchased from the Animal Resources Centre. They were housed in groups of up to four per cage, maintained on a 12/12 h light-dark cycle, and fed standard rodent chow and water *ad libitum*. Peptides diluted in saline containing 0.1% bovine serum albumin (BSA; Sigma-Aldrich) were administered in a volume of 20 μl into the hindpaw by shallow intraplantar injection. Negative-control animals were injected with saline containing 0.1% BSA. Following injection, spontaneous pain behavior events were counted, by a blinded experimenter, from video recordings made for 30 min post injection, as well as 5 min recordings at 1, 2, and 3 h post injection. For analysis of spontaneous pain, a two-way ANOVA with Holm-Šídák's multiple-comparisons test was used to test for differences to negative control over the time course of the experiment and an unpaired *t* test was used to test for differences in the sum of pain behavior counts at 30-min between treated and negative-control animals.

In the experiments testing for amelioration of nocifensive behaviors by TTX, Pm1a and Pm2a (20 pmoles) were administered as above, then at 30 min (once spontaneous nocifensive behaviors had reached near-maximal), mice were injected in the same paw with either saline or TTX (2 μM) and spontaneous nocifensive behavior events were counted from a 5 min video recording by a blinded experimenter.

Experiments involving animals were approved by The University of Queensland Animal Ethics Committee (UQ AEC approval numbers PHARM/526/18 and 2021/AE000448).

### Calcium imaging assay of mammalian sensory neurons

DRG cells were isolated from 5–8-week-old male C57BL/6 mice purchased from the Animal Resources Centre. DRGs were dissociated, then cells plated in Dulbecco’s Modified Eagle’s Medium (Gibco) containing 10% fetal bovine serum (FBS) (Assay matrix) and penicillin/streptomycin (Gibco) on a 96-well poly-D-lysine-coated culture plate (Corning) and maintained overnight. Cells were loaded with Fluo-4 AM calcium indicator, according to the manufacturer’s instructions (Thermo Fisher Scientific). After loading (1 h), the dye-containing solution was replaced with assay solution (Hanks’ balanced salt solution, 20 mM Hepes). Images were acquired at 10× objective at one frame/s (excitation 485 nm, emission 521 nm). Fluorescence corresponding to [Ca^2+^]_*i*_ of ∼250 cells per experiment was monitored in parallel using an Nikon Ti-E deconvolution inverted microscope, equipped with a Lumencor Spectra LED Lightsource. Baseline fluorescence was monitored for 30 s. At 30 s, assay solution was replaced with either assay solution, or assay solution containing TTX (1 μM), Cd^2+^ (100 μM), amiloride (20 μM), or ruthenium red (20 μM), then at 1 min with test peptide (in assay solution ± TTX, Cd^2+^, amiloride, or ruthenium red) and monitored for 2 min before being replaced with assay solution and then KCl (30 mM; positive control). Experiments involving use of mouse tissue were approved by the UQ AEC (approval TRI/IMB/093/17).

### Whole-cell voltage-clamp electrophysiology

HEK293 cells stably expressing the α-subunit of mouse Na_V_1.7, human Na_V_1.6 or Na_V_1.7 plus the β1 subunit (SB Drug Discovery) and Chinese hamster ovary cells stably expressing human Na_V_1.8 plus the β3 subunit in a tetracycline-inducible system (ChanTest), were cultured as previously described (55). Cells were maintained on minimum essential medium supplemented with 10% heat-inactivated FBS, 2 mM L-glutamine in an incubator at 37 °C with 5% CO_2_ and passaged every 3 to 4 days (at 70–80% confluency) using TrypLE Express (Thermo Fisher Scientific).

Whole-cell patch-clamp electrophysiology experiments were performed using a QPatch16 II automated electrophysiology platform (Sophion Bioscience) using single-hole (QPlate 16 with a standard resistance of 2 ± 0.4 MΩ) or multi-hole (QPlate 16X with a standard resistance 0.2 ± 0.04 MΩ, Na_V_1.8 only) plates. Whole-cell currents were filtered at 8 kHz and acquired at 25 kHz and the linear leak was corrected by P/4 subtraction unless stated otherwise.

The extracellular solution (ECS) contained 145 mM NaCl (replaced with 70 mM choline chloride for Na_V_1.7), 4 mM KCl, 2 mM CaCl_2_, 1 mM MgCl_2_, 10 mM Hepes, and 10 mM glucose (pH 7.4; osmolarity, 305 mOsm). The intracellular solution contained 140 mM CsF, 1 mM/5 mM EGTA/CsOH, 10 mM Hepes, and 10 mM NaCl (pH 7.3) with CsOH (osmolarity, 320 mOsm). Peptides were diluted in ECS with 0.1% BSA.

Concentration-response experiments were performed using a holding potential of −90 mV and a 50 ms pulse to −20 mV (+10 mV for Na_V_1.8) every 20 s (0.05 Hz). For washout experiments, peptides were incubated for 200 s before stepwise washout with ECS every 200 s for 30 min. Currents were elicited using a holding potential of −90 mV and a 50 ms pulse to −20 mV every 20 s (0.05 Hz). *I-V* data were obtained with a holding potential of −90 mV followed by a series of 500-ms step pulses that ranged from −110 to +55 mV in 5 mV increments (repetition interval, 5 s) before and after 5 min incubation with peptide. Conductance-voltage curves were obtained by calculating the conductance (*G*) at each voltage (*V*) using the equation *G* = *I*/(*V* – *V*_rev_), where *V*_rev_ is the reversal potential, and they were fitted with the following single or double Boltzmann equations: *I* = *I*_max_/{1 + exp[(*V*_50_ − *V*_m_)/*k*]} or *I* = {*I*_max(a)_/[1+exp({*V*_50(a)_ – *V*_m_}/*k*_(a)_)]} + {*I*_max(b)_/[1 + exp({*V*_50(b)_ – *V*_m_}/*k*_(b)_)]}, where *I*_max_ is the maximal current after normalization to the driving force, *V*_50_ is the half-activation potential, *V*_m_ is the membrane potential, and *k* is the slope factor. Voltage dependence of SSFI was tested using a 10 ms pulse of −20 mV immediately after the 500 ms step above to assess the available non-inactivated channels. For statistical comparison of *G-V* and SSFI curves, a two-tailed paired *t* test was used. Ramp currents were elicited using a slow depolarizing voltage ramp from −100 mV to +20 mV at a rate of 1 mV/ms (120 ms duration).

For human Na_V_1.9 (Icagen, Durham), HEK293 cells stably expressing the α-subunit of hNa_V_1.9 were maintained on minimum essential medium supplemented with 10% heat-inactivated FBS. Whole-cell patch-clamp electrophysiology experiments were performed using a SynchroPatch 384 automated electrophysiology platform (Nanion Technologies). The ECS contained 140 mM NaCl, 4 mM KCl, 2 mM CaCl_2_, 1 mM MgCl_2_, 5 mM glucose, 10 mM Hepes and 100 nM TTX (pH 7.4). The intracellular solution contained 110 mM CsF, 10 mM CsCl, 10 mM NaCl, 10 mM Hepes and 10 mM EGTA (pH 7.2). Peptides were diluted in ECS with 0.1% BSA. Experiments were performed using a holding potential of −130 mV and a 100 ms pulse to −40 mV every 20 s (0.05 Hz) before and after a 4.5 min incubation with 1 μM peptide.

### Insecticidal assay

Blowflies (*L. caesar*; average mass 18 mg) were injected into the ventrolateral thorax with 1 to 2 μl of negative-control solution (water or 5% DMSO) or peptide (in water or 5% DMSO) using a 1 ml Hamilton syringe (1000 Series Gastight, Hamilton Company) with a fixed 29-gauge needle. Flies were assessed for paralysis and/or lethality 1 h post injection. For each assay, up to seven doses of each peptide (*n* = 10 flies per dose) and the appropriate negative control (*n* = 10 flies) were used. Each assay was repeated three times.

### Hemolysis and cytotoxicity assays

Hemolysis and cytotoxicity assays were performed by The Community for Antimicrobial Drug Discovery (CO-ADD). For the hemolytic activity assay, human whole blood was washed three times with three volumes of 0.9% NaCl and then resuspended in 0.9% NaCl to a concentration of 0.5 × 10^8^ cells/ml. Cells were incubated for 1 h at 37 °C with or without the peptide. After incubation, plates were centrifuged at 1000*g* for 10 min to pellet cells and debris and hemolysis determined by measuring the supernatant absorbance at 405 nm. Growth inhibition of HEK293 cells was determined by measuring fluorescence (excitation 530/10 nm and emission 590/10 nm) after the addition of resazurin (25 μg/ml final concentration) and incubation at 37 °C and 5% CO_2_, for an additional 3 h. Percentage growth inhibition was calculated using negative controls (media only) and positive controls (no peptide). Cytotoxic concentration (CC_50_) and hemolytic concentration (HC_50_) values were calculated by curve fitting the inhibition values *versus* log (concentration) using a sigmoidal dose-response function (variable slope), in Pipeline Pilot's dose-response component.

### Statistics

Data were plotted and analyzed using Prism v9.5.1 (GraphPad Software; https://www.graphpad.com/features). Statistical significance was defined as *p* < 0.05. All data are presented as mean ± SEM.

## Data availability

Prepropeptide sequences of MYRTX_1_-Pm1a, MYRTX_1_-Pm2a, MYRTX_1_-Pm3a, MYRTX_1_-Pm4a, MYRTX_1_-Pm5a, MYRTX_1_-Pm6a, MYRTX_1_-Pr1a, MYRTX_1_-Pr2a, MYRTX_1_-Pr2b, MYRTX_1_-Pr2c, MYRTX_1_-Pr2d, MYRTX_1_-Pr3a, MYRTX_1_-Pr3b, MYRTX_1_-Pr4a, MYRTX_1_-Pr4b, MYRTX_1_-Pr4c, MYRTX_1_-Pr5a, MYRTX_1_-Pr6a, MYRTX_1_-Pr6b, MYRTX_1_-Pr7a, MYRTX_1_-Pr8a and MYRTX_1_-Pr8b have been deposited with GenBank, under accessions: OR128458–OR128484, respectively.

*P. maricopa* and *P. rugosus* RNA-seq reads have been deposited in the NCBI sequence read archive under accessions SRR25019486 and SRR25019487, respectively.

The MS proteomics data for *P. maricopa* and *P. rugosus* venom have been deposited to the ProteomeXchange Consortium *via* the PRIDE (56) partner repository with the dataset identifier PXD043773.

## Supporting information

This article contains supporting information.

## Conflict of interest

The authors declare that they have no conflicts of interest with the contents of this article.
