# Supplementary material for: Peptide toxins that target vertebrate voltage-gated sodium channels underly the painful stings of harvester ants
Source: J Biol Chem. 2023 Dec 16;300(1):105577. doi: 10.1016/j.jbc.2023.105577 (PMC10821600; doi:10.1016/j.jbc.2023.105577)
Supplement: Supporting Figure S1 and Table S1 [file mmc1.docx]

**Peptide toxins that target vertebrate voltage-gated sodium channels underly the painful stings of harvester ants.**

Samuel D. Robinson,^1^* Jennifer R. Deuis,^1^ Pancong Niu,^1^ Axel Touchard,^2^ Alexander Mueller,^1,3^ Vanessa Schendel,^1^ Nina Brinkwirth,^4^ Glenn F. King,^1,5^ Irina Vetter,^1,6^ and Justin O. Schmidt^7^

^1^Institute for Molecular Bioscience, The University of Queensland, QLD 4072, Australia.

^2^CNRS, UMR Ecologie des forêts de Guyane – EcoFoG (AgroParisTech, CIRAD, INRAE, Université de Guyane, Université des Antilles), Campus Agronomique, BP 316, 97379 Kourou Cedex, France.

^3^Centro de Investigación Biomédica CENBIO, Universidad UTE, Quito 170527, Ecuador.

^4^Nanion Technologies, Munich 80339, Germany.

^5^Australian Research Council Centre of Excellence for Innovations in Peptide and Protein Science, The University of Queensland, Brisbane, QLD 4072, Australia.

^6^School of Pharmacy, The University of Queensland, Brisbane, QLD 4102, Australia.

^7^Southwestern Biological Institute, Tucson, AZ 85745, USA.

* Corresponding author: Samuel D. Robinson**,** sam.robinson@uq.edu.au

**
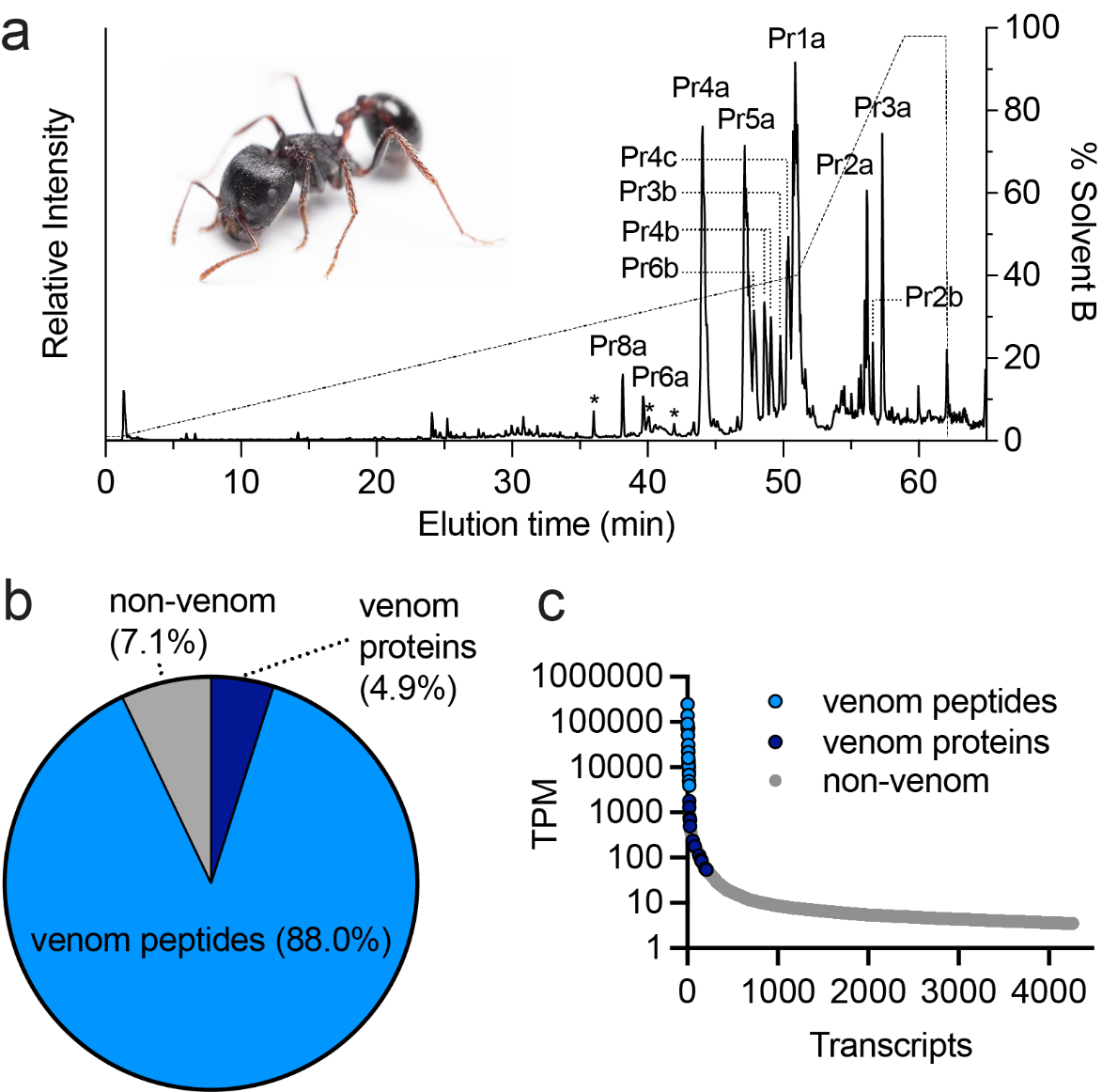
**

**Fig. S1.** The venom composition of *P. rugosus*. (a) Total ion chromatogram from LC-MS/MS analysis of *P. rugosus* venom with labelled peaks corresponding to identified venom peptides. Additional proteins were detected in the reduced, alkylated and trypsin-digested venom sample. Asterisks denote derivatives of identified venom peptides. Inset: Adult female worker caste *P. rugosus* (~9 mm in length). (b) Venom component-encoding transcripts (i.e., those encoding peptides detected in the venom by MS) comprised 92.9% of total transcript expression. Of these, transcripts encoding venom peptides and venom proteins comprised 88.0 and 4.9%, respectively. (c) Venom component-encoding transcripts (highlighted in blue) are found exclusively in the highly expressed portion of the venom-apparatus transcriptome. TPM, transcripts per million.

**Table S1.** Venom components of *P. rugosus*.

|  | TPM | Primary structure |
| --- | --- | --- |
| *Venom peptides:* |  |  |
| Pr1a | 139183 | GLPILAAAVVIPFIHHYLVGKF* |
| Pr2a/c | 106587^†^ | ALPALPLLAFLFSLPAVQHWIEKNWIN* |
| Pr2b/d | 35357^†^ | ALPALPLLAFLFSLPAVQHWVEKNWIN* |
| Pr3a | 51356 | ALPLLPLISLLVSLIPAIKS* |
| Pr3b | 21615 | AVSLILSLIPLASLSPAIKS* |
| Pr4a | 248863 | AS/IDLNEIMKKMRPDLLKMLDDIKTKIQ* |
| Pr4b | 73544 | AS/IDLNEIMKKMGPDLLKMLDDIKTKIQ* |
| Pr4c | 70185 | AS/IDLNEIMKKITPDLLKMLDDIKTKIQ* |
| Pr5a | 75446 | RFLNILKTIGKILLPIIPTVAEKIKEKV* |
| Pr6a | 12462 | LT/DKPGQAKKIRIFDRIPELINWMIVNH |
| Pr6b | 10640 | DKPGQAKKIGIFDRITELVNWLVNH |
| Pr7a*^a^* | 22993 |  |
| Pr8a/b | 11791^†^ | IDPAVLASLV* |
| *Proteins:* |  |  |
| PLA_1_*^b^* | 1371 |  |
| CAPs*^b^* | 929 |  |
| Acid phosphatase*^b^* | 678 |  |
| DPP-4*^b^* | 195 |  |
| PHGPX *^b^* | 90 |  |
| Hyaluronidase *^b^* | 11 |  |

TPM, transcripts per million; *, C-terminal amidation; †, The TPM value is the sum of two paralogous transcripts encoding the same mature peptide. *^a^*, No mature peptide detected in the venom; *^b^*, Detected in reduced, alkylated, trypsin-digested venom sample (> 60% coverage), sequences not shown; PLA_1_, phospholipase-A1; CAP, cysteine-rich secretory protein, insect venom allergen antigen 5, and pathogenesis-related 1 protein; DPP-4, dipeptidyl peptidase IV; PHGPX, Phospholipid-hydroperoxide glutathione peroxidase.
